# Effects of a balance-based exergaming intervention using the Kinect sensor on posture stability in individuals with Parkinson’s disease: a single-blinded randomized controlled trial

**DOI:** 10.1186/s12984-016-0185-y

**Published:** 2016-08-27

**Authors:** Meng-Che Shih, Ray-Yau Wang, Shih-Jung Cheng, Yea-Ru Yang

**Affiliations:** 1 Department of Physical Therapy and Assistive Technology, National Yang-Ming University, 155, Sec 2, Li Nong St., Beitou, Taipei, Taiwan; 2 Department of Neurology, Mackay Memorial Hospital, Taipei, Taiwan

**Keywords:** Balance training, Exergaming, Postural stability, Parkinson’s disease

## Abstract

**Background:**

The present study examined the effects of a balance-based exergaming intervention using the Kinect sensor on postural stability and balance in people with Parkinson’s disease (PD).

**Methods:**

We conducted a subject-blinded, randomized controlled study. Twenty people with PD (Hoehn and Yahr stages I through III) were recruited and randomly assigned to either a balance-based exergaming group (*N* = 10) or a balance training group (*N* = 10) for an 8-week balance training period. Postural stability was assessed using the limits of stability (LOS) and one-leg stance (OLS) tests. Balance was assessed using the Berg Balance Scale (BBS) and the timed up and go (TUG) test. Participants were assessed pre- and post-training.

**Results:**

After training, participants in the balance-based exergaming group showed significant improvements in LOS performance, and in the eyes-closed condition of the OLS test. Both training programs led to improvements in BBS and TUG performance. Furthermore, balance-based exergaming training resulted in significantly better performance in directional control in the LOS test (78.9 ± 7.65 %) compared with conventional balance training (70.6 ± 9.37 %).

**Conclusions:**

Balance-based exergaming training resulted in a greater improvement in postural stability compared with conventional balance training. Our results support the therapeutic use of exergaming aided by the Kinect sensor in people with PD.

**Trial registration:**

ClinicalTrials.gov.NCT02671396

**Electronic supplementary material:**

The online version of this article (doi:10.1186/s12984-016-0185-y) contains supplementary material, which is available to authorized users.

## Background

People with idiopathic Parkinson’s disease (PD) commonly exhibit postural instability during daily activities [1]. PD-related balance impairment is associated with a loss of mobility and increased likelihood of falls, and can cause marked disability [2, 3]. To ameliorate postural instability, techniques using external feedback with cueing or sensory stimuli have been investigated [4, 5]. Several studies suggest that external feedback may initiate other neural pathways and play a significant role in the volitional control of movements for people with PD [6, 7].

Virtual reality (VR) technologies such as exergaming may have therapeutic value in the treatment of postural instability [8–10]. VR is a technology that allows the user to interact directly with a computer-simulated environment [11]. Exergames are computer games that are controlled by body movements. VR and exergaming can provide augmented feedback in real time, while a person performs specific motor tasks [12]. Opportunities for repeated accurate performance can be incorporated into VR and exergaming to enhance motor learning [7, 13]. Moreover, VR games can be effective for retaining participants’ interest and motivation.

A recent meta-analysis suggested that exergaming may provide an appropriate training approach to improve balance and functional mobility in healthy older people [14]. These findings raise the possibility that exergaming might also provide an approach for improving postural instability for people with PD. A previous study examined the effects a 6-week home-based balance training program using the Wii Fit game for a total of 18 training sessions on balance and functional abilities in people with PD, compared with a group of paired healthy participants [15]. Another study investigated the effects of Wii-based training compared with conventional balance training for 7 weeks (a total of 14 training sessions) on activities of daily living in people with PD [16]. Both studies revealed positive effects of exergaming on balance, functional abilities and activities of daily living among people with PD. However, positive effects were found only within groups, with no between-group differences observed in a comparison with the control group. The absence of between-group differences may have resulted from an inability to capture the full-body motion involved in postural control, or the lack of a sufficiently sensitive sensor to accurately measure motion. The shortcomings of the Wii system’s sensors may limit its potential as an effective intervention [17].

A new exergaming system was recently developed using the Kinect sensor. The Kinect sensor is a low-cost device that can provide measurements for most of the main human joints. Previous studies reported that a kinematic measurement method using the Kinect sensor was accurate and reliable for measuring postural control [18, 19]. These findings suggest that the Kinect sensor could provide a useful tool for therapeutic use. However, there has been little research into the therapeutic use of the Kinect sensor to date.

The present study sought to test a therapeutic application of exergaming using the Kinect sensor. We examined the effects of an 8-week balance-based exergaming program developed in our lab, compared with an 8-week period of conventional balance training (16 training sessions), on postural stability and balance in people with PD. We hypothesized that participants who underwent an 8-week balance-based exergaming intervention would demonstrate superior performance on measures of postural stability and balance, compared with those who received balance training.

## Methods

### Participants

Participants were recruited from Mackay Memorial Hospital in Taipei. Outpatients with PD were informed about the study by a neurologist. Eligibility required a diagnosis of idiopathic PD according to the United Kingdom Brain Bank Criteria [20] by the same neurologist. Information on age, gender, the more affected side, and disease duration were obtained through patient interviews and from medical charts. All participants met the following inclusion criteria: (1) Hoehn and Yahr stages I through III, (2) a score of ≥ 24 on the mini-mental state examination, (3) stable medication usage and (4) standing unaided to perform the measurement and training. The exclusion criteria were as follows: (1) histories of other neurological, cardiovascular, or orthopedic diseases affecting postural stability and (2) uncontrolled chronic diseases. In total, 48 individuals were identified as potential participants for this study. Of these, 22 participants gave informed consent and participated in the study.

### Study design

This study was a subject-blinded, randomized controlled trial. The study protocol was approved by the Institutional Review Board of Mackay Memorial Hospital (reference number: 13MMHIS120) and was explained to all participants before their participation. The study was performed in accordance with the Declaration of Helsinki. Block randomization was used to assign participants to either the balance-based exergaming (BE) or the conventional balance training (BT) group. Assignment was performed by an independent person who selected one of a set of sealed envelopes 30 min before the intervention began. Participants in the BE and BT groups received an 8-week balance-based exergaming intervention, and conventional balance training, respectively. Measures of postural stability and functional balance were measured pre- and post-training. The measurement and intervention were conducted with participants in the “on” state, when they were moving freely and easily without dystonia, excessive rigidity or tremor. The data were collected in a university laboratory.

### Intervention

Participants in both groups underwent balance training for 50 min per session, two sessions every week, for 8 weeks. Each training session began with a 10-min warm-up and ended with a 10-min cool-down. Both the warm-up and cool-down periods focused on stretching exercises of the trunk and extremities.

**Fig. 1:**
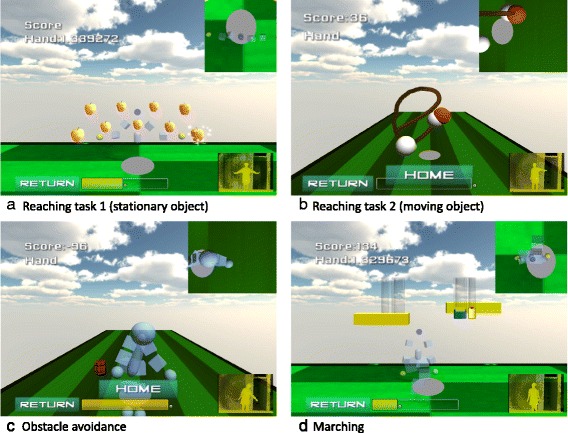
Screen shots of interaction with the exergaming program. Four exergaming programs, Reaching task 1 (**a**), Reaching task 2 (**b**), Obstacle avoidance (**c**) and Marching (**d**), were designed and used for training

Participants in the BE group received a 30-min balance-based exergaming intervention using the Kinect sensor (Microsoft Corporation, Redmond, WA, USA). The Kinect sensor incorporates infrared light and a video camera, which creates a 3D map of the area in front of it. This device provides full-body 3D motion capture. Four exergaming programs were used for training (Fig. 1), designed to incorporate an appropriate level of challenge to match the ability and fitness of people with PD. The first program was called Reaching task 1. In this task, participants were asked to reach toward a stationary target at a given location. The second program was called Reaching task 2. Participants were asked to track a moving object by lengthening the arm and immersing the hand into the object as it flew in 3D space. The third program was called Obstacle avoidance. Participants were instructed to avoid upcoming obstacles that approached from varying directions at random, by moving the body right/left or up/down. The final task was called Marching. Participants were instructed to step alternately without going forward or backward while following dynamic bars that were automatically rising and falling at a predetermined speed and frequency. During the training duration, the challenge level was increased progressively by adjusting the amplitude, frequency, speed, complexity and number of hints. The details of the exergaming programs are shown in Table 1.

**Table 1:** Program of balance-based exergaming intervention

Program	Action	Progression	Motor demand
Reaching task 1	Standing in a given area and reaching toward a stationary target at different heights, depths and in different directions	• Reaching length • Number of targets • Range of distribution • Amount of repetition	• Weight shifting • Challenging limits of stability • Functional transitions
Reaching task 2	Standing in a given area and tracking a moving object while extending arm and immersing the hand into the object as it flew in 3D space	• Speed • Moving range • Pathway pattern • Remembered sequence or course of trajectory	• Weight shifting • Arm coordination • Advance motor planning
Obstacle avoidance	Standing in a given area and preparing to avoid upcoming obstacles that randomly approached from varying directions by moving body sideways or up/down	• Obstacle hitting ratio • Speed • Dual task • Hitting direction	• Quick change strategy • Movement adaption • Agility
Marching	Alternating steps without going forward while following dynamic bars that automatically rose and fell at a predetermined speed and frequency	• Frequency • Gap between steps	• Functional stepping • Leg coordination • Single limb support

Participants in the BT group underwent a 30-min conventional balance training session. The training program included reaching activities, weight-shifting activities and marching activities. The general training protocols used for the BT group were the same as those used for the BE group. The challenge level was increased progressively by changing the base of support, speed, complexity and deprivation of sensory inputs.

### Outcome measures

#### Postural stability

The limits of stability (LOS) and one-leg stance (OLS) tests were used to assess postural stability in this study. Participants were harnessed into a suspension system to prevent falls when performing the tasks. LOS performance was measured using the Smart Balance Master (NeuroCom International Inc., Clackamas, OR, USA) instrument to extract quantitative data [21–24]. The LOS test provides an assessment of the ability to intentionally displace the center of gravity (COG) to the participant’s stability limits without losing balance. In this task, participants were asked to quickly transfer their COG, while standing on stable force plates, toward eight targets spaced at 45° intervals around the COG, represented on a computer monitor. All participants underwent one practice trial followed by one test trial. In the LOS test, we measured reaction time (the time from the presentation of a start cue to the onset of the voluntary shifting of the participant’s COG toward the target position), movement velocity (average speed of COG movement based on the middle 90 % of the distance, measured in degrees per second), end point excursion (percentage of the distance achieved toward a target on the initial movement) and directional control (100 % being a straight line from the center of pressure to the intended target). The validity and reliability of the LOS test in people with neurological disease has been well established [25–27].

The OLS test is an assessment of postural steadiness [15, 28–31]. Participants were asked to cross their arms over the chest, and to stand on either the less or more affected leg, with the other leg raised so that the raised foot was near but not touching the ankle of the stance leg. The assessor timed the OLS test until participants either: (1) uncrossed the arms, (2) moved the stance leg, (3) moved the raised leg touching the floor or the stance leg, (4) opened the eyes on eyes-closed trials or (5) reached a maximum of 30 s. Each participant performed three trials with the eyes open, and three trials with the eyes closed. Data were averaged from the three trials. A previous study found a high degree of reliability (ICC = 0.87) in the OLS test in older adults [32].

#### Functional balance

The Berg Balance Scale (BBS) and the timed up and go (TUG) test were used to assess functional balance. The BBS comprises a set of 14 balance-related tasks, ranging from standing up from a sitting position, to standing on one foot. The degree of success in each task is given a score from zero (unable) to four (independent), and the final measure is the sum of all scores. The highest possible score on the BBS is 56, which indicates excellent balance. The validity and reliability (ICC > 0.95) of BBS scores in people with PD has been established in several studies [33–35]. The TUG test is a mobility test requiring both static and dynamic balance. During the test, the assessors measured the time participants took to rise from a chair, walk 3 meters, turn around, walk back to the chair, and sit down. Each participant performed three trials of the TUG test. Data were averaged from the three trials. The TUG test has previously been found to have high validity and reliability (ICC > 0.87) for assessing balance in people with PD [36, 37].

### Sample size

The sample size calculation was based on a pilot study that tested eight participants at Hoehn and Yahr stages 1 and 2, indicating a difference of 0.2 s between pre- and post-training on reaction time in the LOS test. Based on this difference, a sample size calculation indicated that 20 participants would be sufficient for 85 % power (α = 0.05).

### Statistical analysis

All analyses were performed using the SPSS 20.0 statistical package (SPSS Inc., Chicago, IL, USA). Descriptive statistics were generated for all variables, and distributions of variables were expressed as the mean ± standard deviation. Because of the relatively small number of participants included in the current study (*N* < 30) and since the results of a Shapiro-Wilk test did not allow us to assume that the data were normally distributed, nonparametric tests were employed. Comparison of two groups for general characteristics was made using chi-square or Mann-Whitney *U* test for categorical or continuous variables, respectively. The Friedman test, followed by a post hoc test, was used to determine differences in each dependent variable. The Wilcoxon signed-rank post hoc test was performed for within-group comparisons and the Mann-Whitney U post hoc test was performed for between-group comparisons. The statistical significance was set at *P* ≤ 0.05.

## Results

**Fig. 2:**
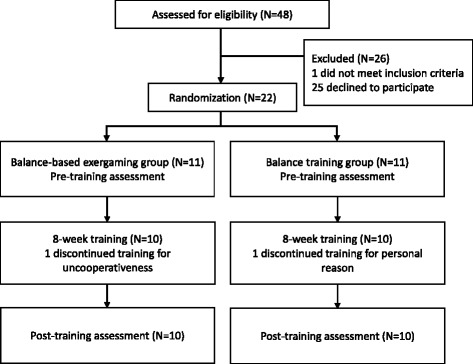
Flowchart of the experimental design

A total of 48 individuals were screened and 22 enrolled between 2013 and 2014. Of these, 11 were assigned to the BT group, and 11 were assigned to the BE group. Of 22 participants, two did not complete the intervention (one in the BT group and one in the BE group). A flow diagram of the study protocol is shown in Fig. 2. The 20 participants who completed the intervention attended all intervention sessions. None of the participants reported any adverse events.

The demographic characteristics of participants in both groups are presented in Table 2. Demographic differences between the two groups were not significant. Moreover, differences in all pre-intervention-selected outcome measures in the two groups were not significant (Table 3).

**Table 2:** Baseline demographics and clinical characteristics of the subjects

	Balance-based exergaming group (*N* = 10)	Balance training group (*N* = 10)	*P*
Age (years)	67.5 ± 9.96	68.8 ± 9.67	0.67
Sex (male/female)	9/1	7/3	0.58
Disease duration (years)	4.03 ± 3.74	5.22 ± 4.85	0.34
Hoehn and Yahr stage	1.6 ± 0.84	1.4 ± 0.52	0.73
Mini-Mental State Examination	27.4 ± 2.59	28.2 ± 1.99	0.40
More affected side (right/left)	8/2	5/5	0.35

Data are presented as the mean ± standard deviation or proportion

**Table 3:** Outcome measures for each group

	Balance-based exergaming group (*N* = 10)		Balance training group (*N* = 10)		Friedman test
	Pre-training	Post-training	Pre-training	Post-training	*P*
Limits of stability					
Reaction time (sec)	0.96 ± 0.33	0.74 ± 0.24*	0.88 ± 0.24	0.79 ± 0.18	<0.001
Movement velocity (deg/sec)	3.37 ± 1.35	3.83 ± 0.97	4.19 ± 1.54	4.57 ± 1.41	0.07
Endpoint excursion (%)	75.2 ± 12.48	84 ± 12.04*	79.7 ± 13.84	81.8 ± 11.37	0.04
Directional control (%)	75.7 ± 8.78	78.9 ± 7.65*^,†^	70.9 ± 10.85	70.6 ± 9.37	0.02
One-leg stance					
Less affected with eyes open (sec)	17.39 ± 12.87	15.16 ± 10.53	9.14 ± 9.63	12.98 ± 11.08	0.47
More affected with eyes open (sec)	15.06 ± 11.23	15.58 ± 11.58	13.72 ± 12.43	14.54 ± 9.65	0.09
Less affected with eyes closed (sec)	3.35 ± 2.85	6.1 ± 8.65*	2.71 ± 2.54	5.31 ± 7.68	0.002
More affected with eyes closed (sec)	3.06 ± 2.55	4.13 ± 2.74	5.88 ± 7.56	6.66 ± 8.41	0.16
Berg Balance Scale	50.9 ± 5.32	53.2 ± 2.86*	50.4 ± 4.79	53 ± 1.89*	0.001
Timed up and go (sec)	9.5 ± 2.45	8.71 ± 1.8*	10.05 ± 4.66	9.18 ± 3.42*	0.007

Data are presented as mean ± standard deviation

*and ^†^are *P* ≤ 0.05 for within-group and between-group comparisons, respectively

The results of the interventions are presented in Table 3. Analysis of selected outcomes using the Friedman test revealed a significant effect of intervention type on reaction time, endpoint excursion and directional control in the LOS test, and in the less affected leg in the eyes-closed condition in the OLS test, the BBS and the TUG test. Within-group post hoc analysis revealed that balance-based exergaming training significantly improved LOS performance (improving reaction time from 0.96 ± 0.33 to 0.74 ± 0.24 s, end point excursion from 75.2 ± 12.48 to 84 ± 12.04 % and directional control from 75.7 ± 8.78 to 78.9 ± 7.65 %) and OLS on the less affected leg in the eyes-closed condition (from 3.35 ± 2.85 to 6.1 ± 8.65 s). Compared with the BT group (70.6 ± 9.37 %), the BE group (78.9 ± 7.65 %) exhibited better performance in directional control of LOS post-training. Functional balance in both groups, as measured by the BBS and the TUG test, was improved significantly post-training compared with pre-training. However, no significant differences were found between groups.

## Discussion

This study produced two main findings: (1) balance-based exergaming training had a greater effect on postural stability compared with conventional balance training; and (2) both training programs improved functional balance in people with PD.

The current study tested two balance training programs with similar training protocols. A recent meta-analysis examined the BBS, postural sway, TUG, and Functional Reach test as measures of postural stability, reporting that exercise therapy is an important treatment option for improving postural stability in people with PD [38]. The findings suggested that exercises containing a balance component were most beneficial in improving postural stability in people with PD [38]. In the current study, we used the LOS and OLS tests to measure postural stability, and the BBS and TUG tests to measure functional balance. The current findings were in line with the findings of Klamroth et al., who reported that balance training was beneficial for performance in the BBS and TUG tests [38]. Our findings revealed that only balance-based exergaming training produced positive effects on LOS and OLS, with particularly strong effects on directional control in LOS. These findings suggest that exergaming training using the Kinect sensor contributed to the beneficial gains we observed. As a therapeutic tool, the Kinect sensor can provide specific motor practice using full-body motion capture, which offers precise real-time information to guide performance and monitor body movement. Previous clinical trials indicated that exergaming programs using the Kinect sensor resulted in accurate capture of movement components [39, 40].

Our results revealed within-group improvements on most measures of postural stability during the exergaming intervention training period. Our exergaming programs involved various balance challenges. This may have contributed to our positive findings, involving actions focused on agility, challenging postural or locomotor-like skills, and reaching away from the base of support. All of these are involved in whole-body movements. In addition, the repetitive, real-time feedback and graded complexity in our exergaming programs may have contributed to the positive effects of training reflected in LOS performance. However, the movement velocity of LOS remained unchanged after exergaming training. Persistent bradykinesia [41] and a choice to focus on improving accuracy rather than faster motor performance among people with PD are possible reasons for our movement velocity findings [21]. The current results also revealed better OLS performance in the eyes-closed condition after exergaming training. A previous study using a Wii-based system reported similar results [15]. Because participants needed to focus on each joint position while carrying out the fine motor plan necessary for many of the tasks in the exergaming training, stimulation of proprioceptive feedback or an improvement in the internal representation of balance may have enhanced OLS performance.

Little evidence is available regarding the minimal clinically important differences in postural stability and balance outcomes in people with PD. Evidence of minimal clinically important differences for LOS and OLS test in PD is lacking. Steffen and Seney reported a minimal detectable change of 5 points on the BBS for people with PD [34]. In the current study, we recorded a 2.45-point improvement after balance training for BBS. The minimally detectable change in TUG performance in people with PD has previously been reported to be 3.5 s [42], which is greater than the 0.83-second improvement observed in the present study. The small but significant changes observed in this study support the therapeutic use of exergaming interventions. However, a greater evidence base is required to support the clinical significance of these results.

Several important characteristics have been identified for useful interventions in PD, suggesting that interventions should be task-specific, progressive, variable in terms of practice, and highly challenging [43, 44]. The exergaming programs designed for the current study involved each of these components. For specificity, the full-body motion capture method can be tailored for the needs of balance strategies. To create an appropriate practice resource and construct the progression and variability of program, we implemented enriched setting parameters by increasing speed, repetition and the addition of tasks. Additionally, the novel motor training gave participants more experience and an opportunity to explore or learn to negotiate the new challenges. Although only directional control in the LOS test showed a significant between-group difference, exergaming training using the Kinect system may provide additional benefits. Participants are able to practice free motions without wearing a sensor that could cause discomfort and inconvenience. Reduced staff intervention and the affordability of the device are important economic benefits of the system. Finally, considering the clinical implications of our findings, the current results suggest that the Kinect system can provide an assistive modality with therapeutic potential as a training tool under the supervision of a therapist.

The current study involved several limitations. First, the sample size was small, limiting the strength to interpret our results. Second, calibration variability was observed during the preparation of each exergaming session. This issue may have influenced the effect of training because calibration was used to normalize each participant’s body information. This formed the basis of the exergaming programs that were tailored for individuals with varying levels of ability. Third, most participants in this study exhibited only mild impairment, and performance at baseline was relatively high. This may have limited the benefits received from training, and the generalizability of our findings to the target population. Finally, the absence of kinematic data meant we were unable to examine spatio-temporal changes in detailed movements.

## Conclusion

The current study revealed that an 8-week period of balance-based exergaming training using the Kinect sensor resulted in a greater improvement of postural stability than conventional balance training. Both exergaming and conventional balance training had positive effects on functional balance. This trial supports the potential therapeutic use of exergaming aided by the Kinect sensor for people with PD. Importantly, the significant changes in BBS and TUG performance observed after both the exergaming and conventional balance training did not reach the minimal detectable change in patients with PD. Further studies on the use of exergaming are needed to verify the clinical implications of these results.

## Additional file

Additional file 1: Dataset. (SAV 6 kb)

## Abbreviations

BBS: Berg Balance Scale
BE: Balance-based exergaming
BT: Balance training
COG: Center of gravity
LOS: Limits of stability
OLS: One-leg stance
PD: Parkinson's disease
TUG: Timed up and go
VR: Virtual reality
